# Cost-Effectiveness of TAVI vs Minimally Invasive SAVR for Low-Risk Aortic Stenosis in Japan

**DOI:** 10.1016/j.jacasi.2026.03.008

**Published:** 2026-04-25

**Authors:** Hiroshi Kawakami, Taisuke Nakayama, Ryo Miyabe, Yasuhisa Nakao, Toru Miyoshi, Kazuhisa Nishimura, Katsuji Inoue, Shuntaro Ikeda, Yoshitsugu Nakamura, Osamu Yamaguchi

**Affiliations:** a Department of Cardiology, Pulmonology, Hypertension and Nephrology, Ehime University Graduate School of Medicine, Toon, Ehime, Japan; b Department of Cardiovascular Surgery, Chiba-Nishi General Hospital, Matsudo, Japan

**Keywords:** aortic stenosis, cost-effectiveness analysis, minimally invasive surgical aortic valve replacement, transcatheter aortic valve implantation

## Abstract

**Background:**

Transcatheter aortic valve implantation (TAVI) and minimally invasive surgical aortic valve replacement (MICS-AVR) are both less invasive options for treating severe aortic stenosis (AS). However, most cost-effectiveness analyses have compared TAVI with conventional full-sternotomy surgery, and the economic value of TAVI relative to MICS-AVR under current surgical practice remains uncertain.

**Objectives:**

This study sought to assess the long-term cost-effectiveness of TAVI compared with MICS-AVR for patients with isolated severe AS from the Japanese health care payer’s perspective.

**Methods:**

A decision analytical model was developed to evaluate long-term clinical and economic outcomes of TAVI and MICS-AVR. Model inputs were derived from randomized trials, meta-analyses, and Japanese national cost data. Incremental cost-effectiveness ratios (ICERs) were calculated in Japanese yen per quality-adjusted life-year (QALY) gained. Deterministic and probabilistic sensitivity analyses tested model robustness, and a scenario analysis reflected outcomes from high-volume Japanese surgical centers. The willingness-to-pay threshold was set at ¥5 million per QALY.

**Results:**

In the base case, TAVI resulted in higher total costs (¥8.45 million vs ¥7.68 million) and slightly greater QALYs (6.55 vs 6.42), yielding an incremental cost-effectiveness ratio of ¥5,885,068 per QALY gained—marginally above the willingness-to-pay threshold, indicating that TAVI was not clearly cost-effective. In the high-volume surgical center scenario, MICS-AVR dominated TAVI, being both less costly and more effective.

**Conclusions:**

TAVI and MICS-AVR showed broadly comparable cost-effectiveness in low-risk patients with isolated severe AS. The relative economic value of TAVI depended strongly on institutional performance and surgical expertise.

Transcatheter aortic valve implantation (TAVI) has revolutionized the treatment of severe aortic stenosis (AS) and is now widely performed across all surgical risk categories. Randomized controlled trials have demonstrated that TAVI provides outcomes comparable or superior to surgical aortic valve replacement (SAVR) in low-risk patients, particularly regarding early recovery, reduced invasiveness, and improved quality of life.^1^, ^2^, ^3^, ^4^, ^5^ Subsequent comparative and economic analyses across broader risk strata have further confirmed these advantages.^6^, ^7^, ^8^ Although TAVI offers clear clinical benefits, it remains substantially more expensive than conventional surgery, prompting extensive cost-effectiveness analyses worldwide.^6^, ^7^, ^8^, ^9^, ^10^, ^11^, ^12^, ^13^, ^14^, ^15^, ^16^ Previous economic analyses consistently show that TAVI is cost-effective in high- and intermediate-risk patients, with incremental cost-effectiveness ratios (ICERs) well below the willingness-to-pay (WTP) thresholds used in various countries.^6^, ^7^, ^8^, ^9^, ^10^^,^^14^^,^^15^

However, evidence in low-risk populations remains limited and less consistent. Recent international studies suggest that although TAVI may be cost-effective in selected low-risk patients, the magnitude of benefit is smaller than in higher-risk cohorts, and results depend strongly on factors such as device cost, long-term durability, and health care setting.^11^, ^12^, ^13^^,^^16^ Importantly, most prior cost-effectiveness studies compared TAVI with conventional full-sternotomy SAVR, and some even included patients undergoing concomitant coronary artery bypass grafting (CABG).^1^^,^^2^ These comparisons may not reflect current surgical practice for isolated AS, where minimally invasive surgical aortic valve replacement (MICS-AVR) has become increasingly common. Advances in surgical techniques—such as mini-sternotomy and right anterior thoracotomy—have achieved outcomes comparable or superior to conventional surgery, with less surgical trauma and faster recovery.^17^, ^18^, ^19^, ^20^, ^21^, ^22^, ^23^, ^24^, ^25^

Therefore, a direct comparison between TAVI and MICS-AVR provides a more relevant and contemporary assessment of their relative clinical and economic value. We aimed to evaluate the cost-effectiveness of TAVI vs MICS-AVR in patients with isolated severe AS using a decision analytical model based on contemporary clinical and economic data.

## Methods

### Study design and perspective

The cost-effectiveness analysis (CEA) was conducted in accordance with internationally and nationally accepted methodologic standards. The overall framework followed the recommendations of the Second Panel on Cost-Effectiveness in Health and Medicine,^26^ ensuring consistent specification of perspective, outcomes, and analytical horizon. Reporting adhered to the Consolidated Health Economic Evaluation Reporting Standards statement^27^ to ensure transparency and reproducibility.

Country-specific methodologic requirements were incorporated based on the Japanese Guideline for Economic Evaluation of Healthcare Technologies.^28^ Analyses were performed from the Japanese health care payer’s perspective, with both costs and health outcomes discounted at an annual rate of 2% in the base case. Results were expressed as ICERs, defined as cost per quality-adjusted life-year (QALY) gained.

The model used a base-case 10-year time horizon with a monthly cycle length to capture both short-term perioperative outcomes and long-term valve durability. This time frame reflects the average survival and prosthetic valve lifespan of older Japanese patients with severe AS. A half-cycle correction was applied to avoid bias in time-dependent transitions. Annualized event rates were derived from published survival data, and transitions between health states were modeled using parametric survival functions where applicable.

Uncertainty was assessed through deterministic 1-way sensitivity analyses and probabilistic sensitivity analyses using 10,000 Monte Carlo simulations. Scenario analyses examined alternative assumptions, including variations in surgical performance and valve durability. The WTP threshold was set at ¥5 million per QALY, consistent with prior Japanese studies,^29^ and ¥7 million per QALY was considered in sensitivity analyses to reflect severity-adjusted valuations reported in Japan.^30^ The study was approved by the Research Ethics Committee of the Ehime University Graduate School of Medicine (number 2302011).

### Decision model

The analysis focused on low-risk patients with isolated severe AS, typically in their mid-70s, undergoing valve replacement. The interventions compared were TAVI and MICS-AVR. To ensure a pure comparison, patients undergoing concomitant procedures (eg, CABG, additional valve interventions) were excluded.

**Figure 1:**
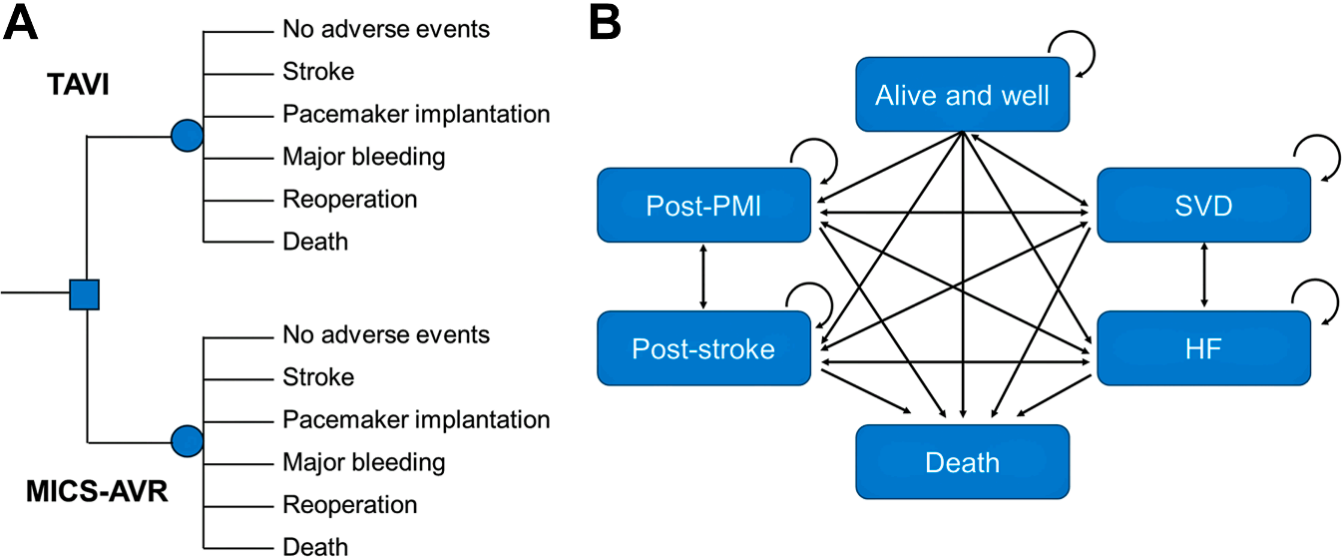
Model Structure for the Cost-Effectiveness Analysis (A) Decision tree representing the acute (periprocedural) phase for TAVI and MICS-AVR. (B) Markov model illustrating transitions between postprocedural health states over time, including “alive and well,” post-PMI, poststroke, HF, SVD, and death. HF = heart failure; MICS-AVR = minimally invasive surgical aortic valve replacement; SVD = structural valve deterioration; TAVI = transcatheter aortic valve implantation.

The model structure was developed based on previously published cost-effectiveness models in valvular heart disease and was adapted to reflect contemporary Japanese clinical practice. The final structure was determined through consensus among experienced TAVI cardiologists and MICS-AVR surgeons practicing in Japan. It consisted of 2 components (Figure 1): an acute phase (0-30 days) represented by a decision tree, and a chronic phase (≥1 month) modeled using a Markov process over 10 years in the base-case analysis. To explore the potential impact of longer-term durability and reintervention strategies in low-risk patients, we additionally performed an exploratory extension of the analytical time horizon to 15 years using identical structural assumptions, transition probabilities, and discounting methods.

During the acute phase, patients could experience perioperative events including stroke, permanent pacemaker implantation (PMI), major bleeding, reoperation, or death. Major bleeding was assumed to be fully managed within the 30-day period without persistent health-state separation.

At the completion of the acute phase, patients entered the chronic Markov model according to their perioperative outcomes. Patients without major complications transitioned to the “alive and well” state. Patients who experienced stroke entered the “poststroke” state, and those undergoing PMI entered the “post-PMI” state. Patients requiring reoperation transitioned to “alive and well” if they survived or to the absorbing “death” state if the event was fatal. Patients who died during the acute phase entered the absorbing “death” state.

The chronic phase included the following health states: “alive and well,” “poststroke,” “post-PMI,” “heart failure,” “structural valve deterioration,” and “death.” Transitions occurred between clinically plausible states, including late-onset complications. Event rates were derived from published data, annualized, and converted into monthly transition probabilities. State-specific costs and utilities were applied according to health status over time, and initial postacute health states determined subsequent long-term clinical and economic trajectories.

### Transition probabilities

Event probabilities were derived from randomized controlled trials, meta-analyses, and large international registries (Table 1).^3^, ^4^, ^5^^,^^18^^,^^21^, ^22^, ^23^, ^24^, ^25^^,^^31^, ^32^, ^33^, ^34^, ^35^ The base-case analysis used international data for both arms, whereas outcomes for MICS-AVR were extracted specifically from studies evaluating minimally invasive approaches (mini-sternotomy or right anterior thoracotomy) in low- to intermediate-risk patients, rather than conventional full-sternotomy cohorts.

**Table 1:** Event Probabilities for the Base-Case Model

	TAVI	MICS-AVR	Distribution	Ref. #
Perioperative (30-d) complications				
Stroke, %	0.6 (0.5-1.2)	1.2 (0.6-2.0)	Beta	^3^^,^^4^^,^^18^^,^^21^, ^22^, ^23^, ^24^, ^25^
Permanent pacemaker implantation, %	12.0 (6.5-17.4)	5.0 (4.0-6.1)	Beta	^3^^,^^4^^,^^18^^,^^21^, ^22^, ^23^, ^24^, ^25^^,^^35^
Major bleeding, %	2.4 (2.4-3.6)	4.3 (3.0-6.0)	Beta	^4^^,^^18^^,^^21^, ^22^, ^23^, ^24^, ^25^
Reoperation, %	2.0 (1.0-3.0)	4.3 (3.0-6.0)	Beta	^3^^,^^4^^,^^18^^,^^21^, ^22^, ^23^, ^24^, ^25^
Death, %	0.8 (0.5-1.3)	1.0 (0.7-1.9)	Beta	^3^^,^^4^^,^^18^^,^^21^, ^22^, ^23^, ^24^, ^25^
Chronic phase (annual) events				
All-cause death (per year), %	1.8 (1.5-2.2)	1.8 (1.5-2.2)	Beta	3, 4, 5^,^^18^^,^^21^, ^22^, ^23^, ^24^, ^25^
Heart failure hospitalization (per year), %	1.6 (1.3-1.9)	3.4 (3.1-3.7)	Beta	^3^^,^^4^^,^^18^^,^^21^^,^^22^^,^^25^
Permanent pacemaker implantation (per year), %	0.50 (0.30-0.80)	0.30 (0.10-0.50)	Beta	^3^^,^^4^^,^^18^^^,^^^21^^,^^22^^,^^25^^,^^33^, ^34^, ^35^
Stroke (per year), %	0.64 (0.50-0.85)	0.83 (0.60-1.10)	Beta	^3^^,^^4^^,^^18^^,^^21^^,^^22^^,^^25^
SVD (per year), %	1.7 (1.3-2.0)	3.5 (2.5-4.5)	Beta	3, 4, 5^,^^18^^,^^21^^,^^22^^,^^25^^,^^31^^,^^32^
Reintervention for SVD (per year), %	0.63 (0.30-1.10)	0.55 (0.20-0.90)	Beta	3, 4, 5^,^^18^^,^^21^^,^^22^^,^^25^^,^^31^^,^^32^

Values are mean (range).

MICS-AVR = minimally invasive surgical aortic valve replacement; SVD = structural valve deterioration; TAVI = transcatheter aortic valve implantation.

Perioperative and chronic event probabilities were modeled separately, with acute risks applied in the first cycle and chronic risks in subsequent annual cycles. Each model parameter was assigned a mean value and plausible range based on published evidence. All parameter values, ranges, and references are summarized in Table 1. Annualized event rates were calculated as r = −ln(S)/t, where S denotes the reported survival probability at time t (in years), and converted into monthly transition probabilities. This conversion assumes an exponential survival distribution, implying a constant hazard over time.

When only ranges were available, these were assumed to approximate 95% CIs, and SDs were estimated as (high − low)/3.92. Beta distributions were parameterized by matching the reported means and variances.

In the chronic phase, transition probabilities were specified at the event level rather than at the state-to-state level. In the absence of robust state-specific transition data applicable to low-risk populations, identical annual event risks were applied across relevant health states. Accordingly, long-term transition probabilities were modeled as independent of prior perioperative events, whereas state-specific costs and utilities were applied according to health status.

### Costs

All costs were estimated from the Japanese health care payer’s perspective and expressed in 2025 Japanese yen, with a 2% annual discount rate applied in the base case. The analysis included procedural, complication-related, and chronic phase costs, such as outpatient follow-up, medications, heart failure management, and pacemaker care. One-time costs were applied for heart failure hospitalization, stroke, and reintervention due to structural valve deterioration. Cost parameters were primarily obtained from the official reimbursement schedules and drug price standards published by the Japanese Ministry of Health, Labour and Welfare (2024-2025).^36^ Procedure-related costs were informed by the Diagnosis Procedure Combination reimbursement framework, which covers the most hospitals performing TAVI and cardiac surgery in Japan. These estimates were supplemented and cross validated using large-scale Japanese economic analyses.^37^^,^^38^ Additional cost inputs were derived from published Japanese studies.^39^, ^40^, ^41^ Full parameter sets and references are provided in Table 2.

**Table 2:** Cost Parameters Applied in the Base-Case, Sensitivity, and Scenario Analyses

	TAVI	MICS-AVR	Distribution	Ref. #
Perioperative costs				
Device and procedural cost	4,346,090 (3,476,872-5,215,308)	1,478,300 (1,182,640-1,773,960)	Gamma	^37^^,^^38^
Procedure fee	390,600 (390,600-390,600)	855,000 (855,000-855,000)	Fixed	^36^
Incremental costs of procedural complications				
Other hospitalization cost	1,315,720 (1,052,576-1,578,864)	2,606,140 (2,084,912-3,127,368)	Gamma	^37^
In-hospital death	300,000 (150,000-600,000)	300,000 (150,000-600,000)	Gamma	^37^
Stroke	1,600,000 (1,200,000-2,000,000)	1,600,000 (1,200,000-2,000,000)	Gamma	^39^^,^^40^
Pacemaker implantation	1,200,000 (800,000-1,500,000)	1,200,000 (800,000-1,500,000)	Gamma	^36^
Major bleeding	400,000 (200,000-600,000)	400,000 (200,000-600,000)	Gamma	^37^
Reoperation	3,000,000 (2,500,000-3,500,000)	3,000,000 (2,500,000-3,500,000)	Gamma	^36^^,^^37^
Annual costs for chronic phase (per year)				
Follow-up cost	40,000 (30,000-60,000)	40,000 (30,000-60,000)	Gamma	^36^
Medication	100,000 (60,000-200,000)	100,000 (60,000-200,000)	Gamma	^36^
Pacemaker follow-up	68,000 (65,000-72,000)	68,000 (65,000-72,000)	Gamma	^36^
Poststroke care	500,000 (300,000-1,000,000)	500,000 (300,000-1,000,000)	Gamma	^39^^,^^40^
Heart failure follow-up	40,000 (20,000-80,000)	40,000 (20,000-80,000)	Gamma	^36^
One-time cost for chronic phase events (per events**)**				
Heart failure hospitalization	1,300,000 (1,100,000-1,500,000)	1,300,000 (1,100,000-1,500,000)	Gamma	^41^
Pacemaker implantation	1,200,000 (800,000-1,500,000)	1,200,000 (800,000-1,500,000)	Gamma	^36^
Stroke	1,600,000 (1,200,000-2,000,000)	1,600,000 (1,200,000-2,000,000)	Gamma	^39^^,^^40^
Reintervention for SVD	4,940,000 (4,420,000-5,480,000)	5,500,000 (4,900,000-6,200,000)	Gamma	36, 37, 38

All costs are expressed in Japanese yen and were primarily derived from the official national reimbursement schedule and Diagnosis Procedure Combination-based analyses. Estimates were cross validated using registry data and published Japanese cost studies (MHLW 2024-2025, JROAD, and other large-scale sources).

JROAD = Japanese Registry of All Cardiac and Vascular Diseases; MHLW = Ministry of Health, Labour and Welfare; other abbreviations as in Table 1.

### Utility

Health state utilities were derived from published studies on severe AS and heart failure. Baseline utility was set at 0.62, increasing to 0.75 to 0.78 within 1 year after valve replacement and stabilizing at 0.80 thereafter. Beyond 1 year, postoperative utility was assumed to remain stable due to the absence of robust long-term longitudinal utility data in contemporary low-risk populations. Utility decrements were applied for adverse events: −0.20 for chronic stroke sequelae, −0.10 for acute stroke (1 cycle), −0.18 for heart failure hospitalization, −0.08 for chronic heart failure, −0.02 for PMI, and −0.04 for structural valve deterioration. All utility parameters were derived from published literature, including the PARTNER (Placement of Aortic Transcatheter Valves) trial quality-of-life substudy as a representative source.^42^ Complete parameter sets and additional references are provided in Supplemental Table 1.

### Sensitivity analyses

Both deterministic and probabilistic sensitivity analyses were conducted to assess the impact of parameter uncertainty on model outcomes. In deterministic analyses, 1-way sensitivity analyses were first performed by varying each parameter independently to identify key drivers of the results. Additionally, 2-way sensitivity analyses were conducted to examine the combined influence of selected pairs of parameters—primarily procedural costs and event probabilities—on the ICER. In addition to parameter-based deterministic analyses, we conducted a structural sensitivity analysis examining the impact of the analytical time horizon. Specifically, to assess potential truncation bias associated with the 10-year base-case horizon, the model was extended to 15 years while maintaining identical structural assumptions, transition probabilities, and discounting methods. Furthermore, we conducted additional deterministic sensitivity analyses by varying the annual discount rate between 0% and 4% to evaluate the robustness of the ICER to alternative discounting assumptions.

Parameter ranges are summarized in Tables 1 and 2 and Supplemental Table 1. When 95% CIs were available, they were used as bounds; otherwise, parameters were varied by ±25% of base-case value or within minimum and maximum values reported in the literature. Tornado diagrams were generated to illustrate the relative importance of each parameter, and 2-way sensitivity plots were used to visualize the threshold relationships between costs and effectiveness.

In probabilistic sensitivity analyses, 10,000 Monte Carlo simulations were performed to evaluate the overall effect of joint parameter uncertainty in probabilities, utilities, and costs. Appropriate probability distributions were assigned according to parameter type: beta for probabilities and utilities, gamma for costs, and log-normal or triangular for other parameters (Tables 1 and 2, Supplemental Table 1). Parameters were sampled independently across simulations. Although clinical outcomes such as mortality, heart failure hospitalization, and health-related quality of life may be correlated in practice, robust empirical covariance data were not available to parameterize these relationships. Cost-effectiveness acceptability curves derived from these simulations were used to represent the probability that each strategy would be considered cost-effective across a range of WTP thresholds.

### Scenario analysis

Scenario analyses were performed to examine the robustness of the base-case results under alternative clinical assumptions. These analyses also explored a scenario informed by outcomes from Japanese high-volume centers to assess how performance in experienced domestic programs might influence cost-effectiveness. Specifically, the MICS-AVR arm was reparameterized using outcomes from Japanese high-volume centers where minimally invasive valve surgery is routinely performed by experienced surgeons. Point estimates were recalibrated based on published data from Japanese high-volume studies. Uncertainty ranges were defined with reference to both these domestic reports and the base-case sources to ensure realistic variability and to avoid overly narrow assumptions regarding procedural consistency. The event probabilities applied in this scenario are summarized in Supplemental Table 2. For the TAVI arm, base-case probabilities were retained because contemporary Japanese registry data suggest broadly comparable outcomes to international reports. The model structure, costs, and utility parameters were maintained as in the base case to isolate the effect of procedure-specific outcomes on cost-effectiveness.

## Results

### Base-case analysis

**Figure 2:**
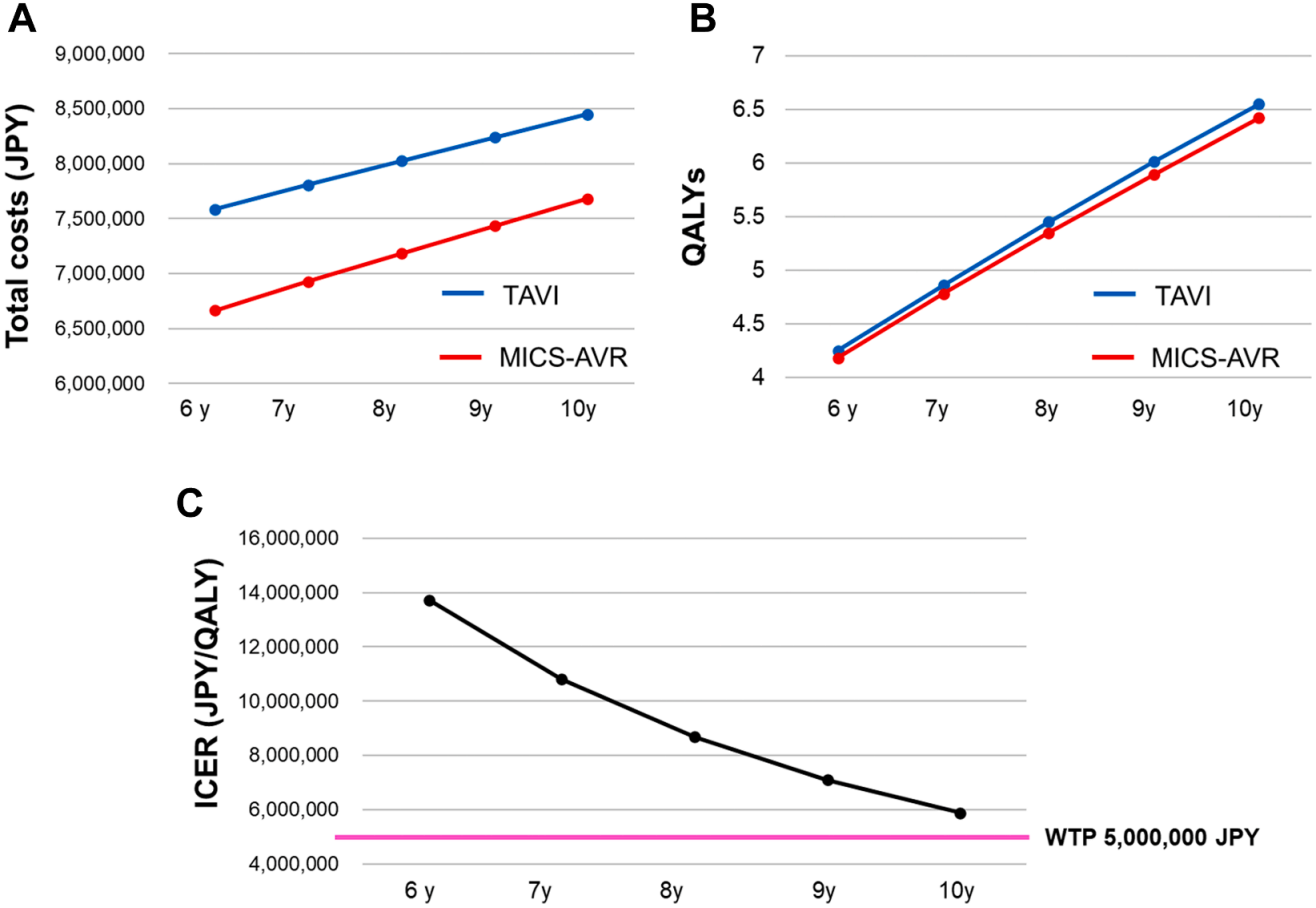
Temporal Trends in Cost-Effectiveness Outcomes Over a 10-Year Horizon (A) Cumulative total costs, (B) total QALYs, and (C) trajectories of the ICER for TAVI and MICS-AVR. The ICER for TAVI decreased from approximately ¥14 million per QALY at year 6 to <¥6 million at year 10, approaching the Japanese WTP threshold of ¥5 million per QALY. ICER = incremental cost-effectiveness ratio; JPY = Japanese yen; QALY = quality-adjusted life-year; WTP = willingness-to-pay; other abbreviations as in Figure 1.

Over the 10-year analytical horizon, total costs were higher for TAVI (¥8,451,136) than for MICS-AVR (¥7,681,154), whereas total QALYs were slightly greater with TAVI (6.55 vs 6.42 QALYs). The resulting ICER was ¥5,885,068 per QALY gained (Table 3). At Japan’s standard WTP threshold of ¥5 million per QALY, TAVI did not meet the cost-effectiveness criterion; however, it became cost-effective when a higher threshold of ¥7 million per QALY was applied. Temporal trend analysis (Figure 2) demonstrated a steady decline in ICER values over time—from approximately ¥14 million per QALY at year 6 to <¥6 million per QALY at year 10—indicating that extended survival and accumulated QALY gains progressively improved the economic efficiency of TAVI relative to MICS-AVR. The complete year-by-year results for total costs, QALYs, and ICERs from years 1 through 10 are provided in Supplemental Table 3.

**Table 3:** Base-Case Cost-Effectiveness Results (10-Year Horizon)

Strategy	Total Costs (JPY)	Incremental Costs (JPY)	Total QALYs	Incremental QALYs	ICER (JPY/QALY Gained)
MICS-AVR	7,681,154		6.42		
TAVI	8,451,136	769,982	6.55	0.13	5,885,068

ICER = incremental cost-effectiveness ratio; JPY = Japanese yen; QALY = quality-adjusted life year; other abbreviations as in Table 1.

### Deterministic sensitivity analyses

**Figure 3:**
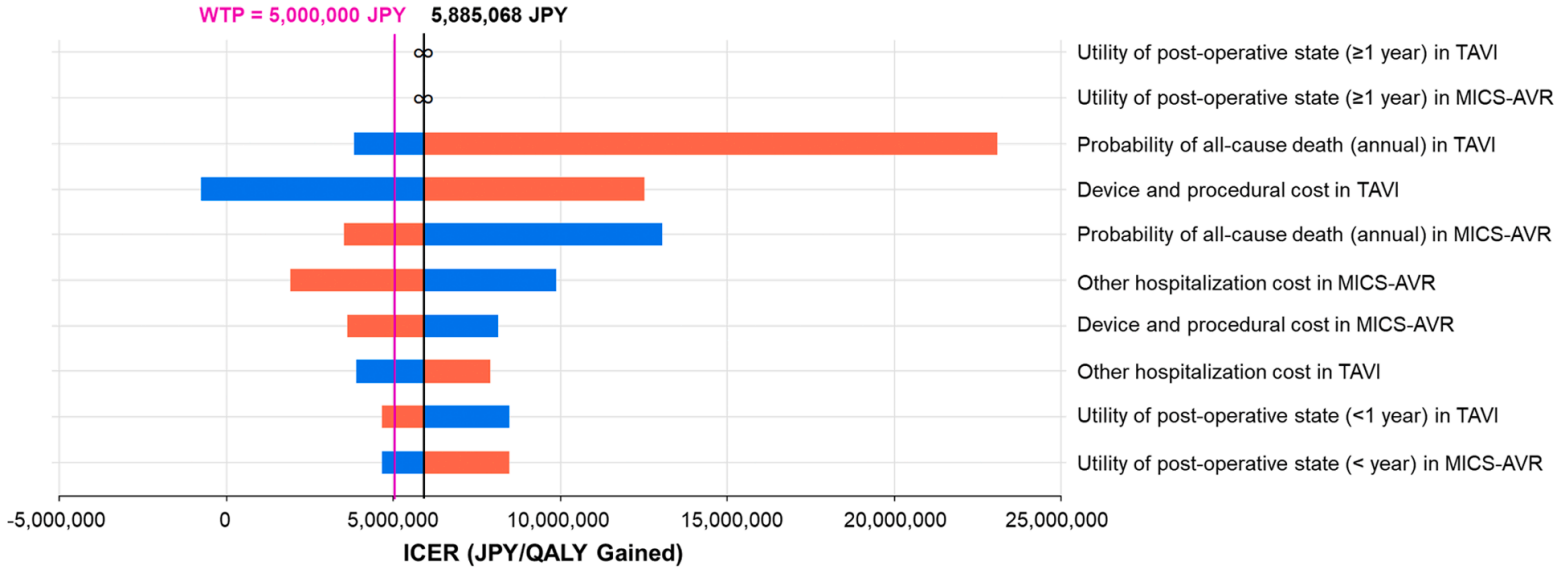
Deterministic Sensitivity Analyses in the Base-Case Analysis Tornado diagram shows the impact of key model parameters on the ICER of TAVI vs MICS-AVR. Blue bars represent the ICER when each parameter was set at the lower bound of its predefined range, whereas red bars represent the ICER when the parameter was set at the upper bound. Postoperative utility beyond 1 year had the greatest influence, followed by all-cause mortality during the chronic phase and device/procedural costs. The vertical lines indicate the base-case ICER (black) and the Japanese WTP threshold of ¥5 million per QALY (magenta). Abbreviations as in Figures 1 and 2.

In the 1-way sensitivity analyses (Figure 3), the postoperative utility beyond 1 year exerted the greatest influence on the ICER, followed by the annual all-cause mortality during the chronic phase and device or procedural costs. These findings indicate that long-term quality of life and survival assumptions were the primary drivers of cost-effectiveness, whereas variations in other parameters—such as heart failure hospitalization, stroke, or structural valve deterioration—had only minor effects on model outcomes.

**Figure 4:**
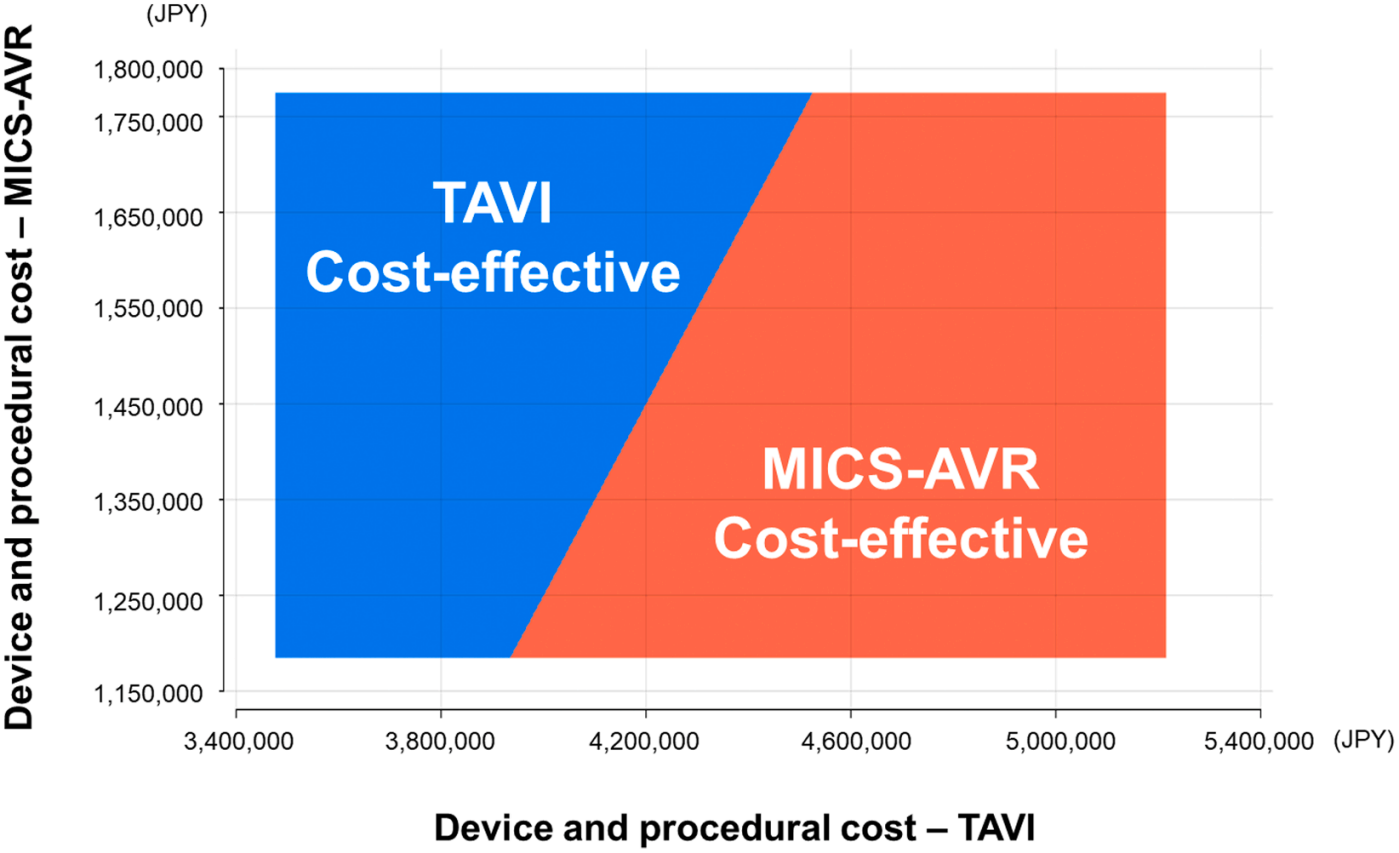
Two-Way Deterministic Sensitivity Analysis 2-dimensional plot illustrates the cost-effectiveness regions according to simultaneous variations in device and procedural costs for TAVI and MICS-AVR. As the cost difference between the 2 strategies narrowed, the cost-effectiveness frontier shifted in favor of TAVI, emphasizing the impact of cost containment on its economic value. Abbreviations as in Figures 1 and 2.

Two-way sensitivity analyses (Figure 4) simultaneously varied device and procedural costs for both strategies. As the cost difference between TAVI and MICS-AVR narrowed, the cost-effectiveness frontier shifted toward TAVI, indicating that further reductions in device or procedural costs would enhance its economic value within Japan’s WTP threshold. Taken together, these results suggest that the cost-effectiveness of TAVI in low-risk populations is primarily determined by long-term utility, survival, and procedural cost assumptions.

In the time-horizon sensitivity analysis extending the model to 15 years, ICER values continued to decline over time, further improving the cost-effectiveness profile of TAVI relative to MICS-AVR (Supplemental Table 3). In additional sensitivity analyses varying the annual discount rate from 0% to 4%, the ICER ranged from approximately ¥5.36 million to ¥6.41 million per QALY, remaining within a similar range compared with the base-case discount rate of 2%.

### Probabilistic sensitivity analyses

In the probabilistic sensitivity analysis based on 10,000 Monte Carlo simulations, TAVI was dominant in 5.5% of simulations, whereas MICS-AVR was dominant in 34.9%. In the remaining simulations, TAVI was more effective but more costly than MICS-AVR, reflecting a trade-off scenario consistent with the base-case incremental results.

**Figure 5:**
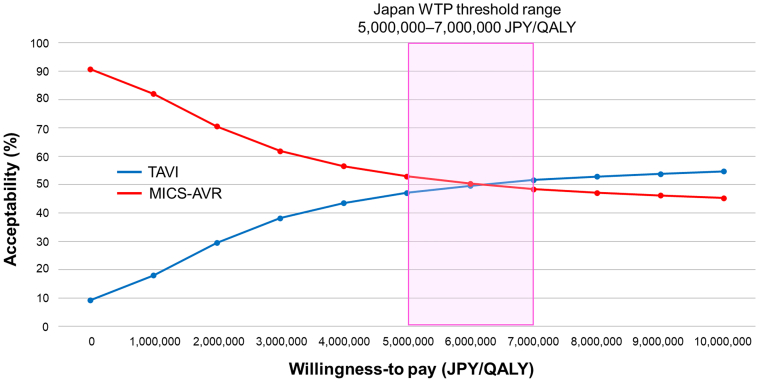
Cost-Effectiveness Acceptability Curves Acceptability curves illustrate the probability of each strategy being cost-effective across a range of WTP thresholds. The curves intersected at approximately ¥6 million per QALY, indicating that TAVI was not cost-effective at the conventional Japanese threshold of ¥5 million per QALY but achieved cost-effectiveness under a higher threshold of ¥7 million per QALY. Abbreviations as in Figures 1 and 2.

Cost-effectiveness acceptability curves demonstrated that the likelihood of TAVI being cost-effective increased gradually with higher WTP thresholds (Figure 5). The acceptability curves for TAVI and MICS-AVR intersected at approximately ¥6 million per QALY, indicating that TAVI was not cost-effective at the conventional Japanese threshold of ¥5 million per QALY but became cost-effective at ¥7 million per QALY.

**Figure 6:**
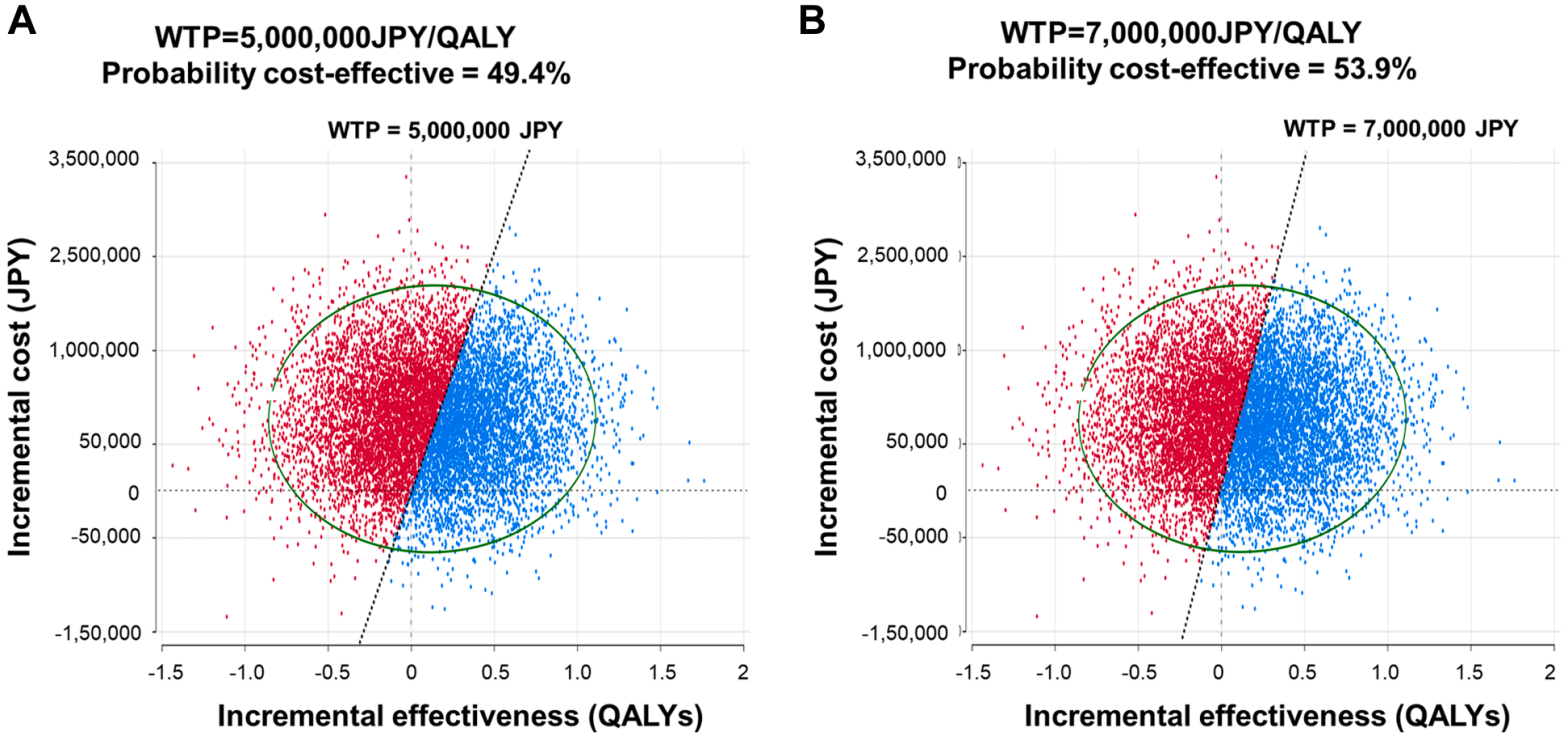
Probabilistic Sensitivity Analysis Results (A and B) Incremental cost-effectiveness scatterplots derived from 10,000 Monte Carlo simulations at WTP thresholds of ¥5 million (A) and ¥7 million (B) per QALY. Each dot represents 1 simulation. Blue dots indicate simulations in which transcatheter aortic valve implantation was cost-effective at the specified WTP threshold, whereas red dots indicate simulations in which it was not. The proportion of simulations in which transcatheter aortic valve implantation was cost-effective was 49.4% at a WTP of ¥5 million per QALY and 53.9% at ¥7 million per QALY. Abbreviations as in Figure 2.

Specifically, the probability that TAVI was cost-effective was 49.4% at a WTP of ¥5 million per QALY and 53.9% at ¥7 million per QALY (Figure 6). Consistent with base-case results, TAVI was associated with higher costs and marginally greater health benefits compared with MICS-AVR, yielding an ICER close to the Japanese WTP threshold and suggesting borderline cost-effectiveness under current economic conditions.

### Scenario analysis

In the scenario assuming MICS-AVR was performed by experienced surgeons in high-volume Japanese centers, TAVI remained more costly but was slightly less effective than surgical intervention (Supplemental Table 4). The resulting ICER indicated that MICS-AVR dominated TAVI, with lower total costs (¥6,670,307 vs ¥8,451,136) and higher total QALYs (6.60 vs 6.55). Probabilistic sensitivity analyses using 10,000 Monte Carlo simulations (Supplemental Figures 1 and 2) demonstrated that MICS-AVR had a higher probability of being cost-effective across the full range of WTP thresholds. At a WTP of ¥5 million per QALY, the probability of MICS-AVR being cost-effective was 71.5%, increasing to 77.6% at ¥7 million per QALY.

These findings suggest that, under conditions of optimal surgical expertise and procedural efficiency, MICS-AVR becomes the economically preferred strategy in low-risk patients, whereas TAVI does not achieve cost-effectiveness within conventional Japanese WTP thresholds.

## Discussion

This study examined the cost-effectiveness of TAVI compared with MICS-AVR in patients with isolated severe AS at low surgical risk. In the base-case analysis over a 10-year analytical horizon, TAVI incurred higher costs and yielded marginally greater health benefits, resulting in an ICER close to Japan’s conventional WTP threshold. Sensitivity analyses indicated that long-term utility was the most influential determinant of cost-effectiveness, followed by surgical and device-related costs. In an extended time-horizon sensitivity analysis to 15 years, ICER values continued to decline over time, further supporting the stability of the base-case conclusions. In the scenario reflecting high-volume centers with experienced surgeons, the cost-effectiveness balance shifted toward MICS-AVR; however, this likely reflects the benefit of optimal surgical performance rather than an inherent disadvantage of TAVI. Taken together, these findings suggest that the economic value of TAVI relative to MICS-AVR is highly context-dependent. The overall study design, principal findings, and policy implications are summarized in the Central Illustration.

**Central Illustration:**
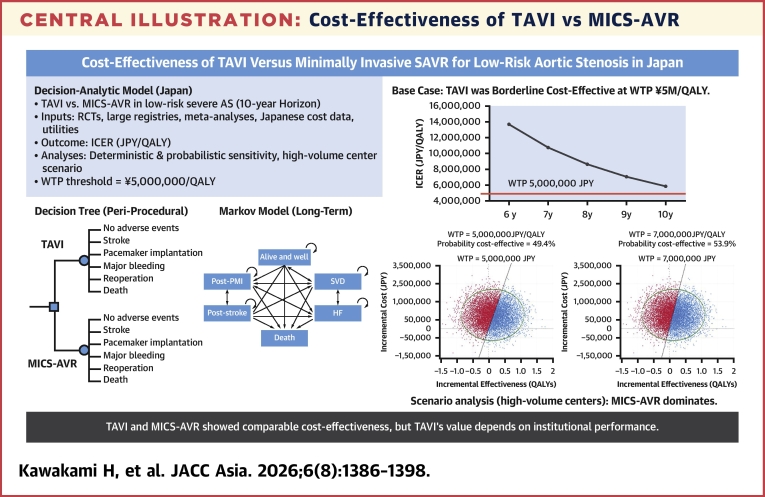
Cost-Effectiveness of TAVI vs MICS-AVR Summary of the decision analytical model and principal findings. A hybrid model consisting of a periprocedural decision tree and a long-term Markov process was used to compare TAVI and MICS-AVR in low-risk AS over a 10-year horizon. In the base-case analysis, TAVI demonstrated slightly higher QALYs at greater cost, resulting in a borderline ICER near the Japanese WTP of ¥5 million per QALY. Probabilistic sensitivity analysis indicated substantial decision uncertainty. In a scenario reflecting high-volume surgical centers, MICS-AVR became economically dominant. Overall, relative cost-effectiveness depends strongly on postoperative utility and institutional performance. AS = aortic stenosis; HF = heart failure; ICER = incremental cost-effectiveness ratio; JPY = Japanese yen; MICS-AVR = minimally invasive surgical aortic valve replacement; PMI = permanent pacemaker implantation; QALY = quality-adjusted life-year; RCT = randomized controlled trial; SVD = structural valve deterioration; TAVI = transcatheter aortic valve implantation; WTP = willingness-to-pay.

### Comparison with previous studies on TAVI vs conventional SAVR

Several CEAs have compared TAVI with conventional SAVR across different surgical risk categories. In high-risk and intermediate-risk populations, landmark CEAs based on the PARTNER and SURTAVI (Surgical Replacement and Transcatheter Aortic Valve Implantation) trials consistently demonstrated that TAVI was cost-effective, or even dominant, compared with SAVR, largely due to shorter hospital stays and reduced procedural invasiveness.^6^, ^7^, ^8^, ^9^

For low-risk patients, however, the results have been less consistent. Analyses derived from the PARTNER 3 (Placement of Aortic Transcatheter Valves 3 trial, a randomized trial comparing transcatheter aortic valve implantation with surgical aortic valve replacement in low-risk patients) and Evolut Low Risk (Evolut Low Risk trial, a randomized trial comparing self-expanding transcatheter aortic valve implantation with surgical aortic valve replacement in low-risk patients) trials reported ICERs near or slightly above conventional WTP thresholds, suggesting that the economic advantage of TAVI diminishes as surgical risk decreases.^11^, ^12^, ^13^ In Japan, several CEAs conducted after the introduction of TAVI reimbursement generally supported its cost-effectiveness in high- and intermediate-risk cohorts, whereas evidence in low-risk populations remains limited.^14^, ^15^, ^16^

Notably, most Japanese and international analyses compared TAVI with conventional full-sternotomy SAVR and frequently included patients undergoing concomitant CABG. Such comparisons may not accurately represent current clinical practice, in which MICS-AVR has become increasingly common for isolated AS.^17^, ^18^, ^19^, ^20^ The present study addressed this evidence gap by directly comparing TAVI with MICS-AVR, offering a more contemporary evaluation of 2 minimally invasive strategies. Our findings demonstrated that their cost-effectiveness was broadly comparable under appropriate clinical conditions.

### Clinical performance and potential advantages of MICS-AVR

MICS-AVR, performed via approaches such as mini-sternotomy or right anterior thoracotomy, has been increasingly adopted as an alternative to conventional full-sternotomy aortic valve replacement in patients with isolated AS. A systematic review and meta-analysis found no increase in mortality compared with full sternotomy, whereas intensive care unit (ICU) stay, overall hospitalization, ventilation time, and early blood loss were all reduced with ministernotomy.^17^ In a propensity-matched comparison, Glauber et al^18^ reported that right anterior minithoracotomy aortic valve replacement achieved equivalent perioperative mortality and complication rates to full sternotomy, with lower transfusion requirements and shorter hospital stays. Recent large-scale, propensity-matched analyses have confirmed these benefits, demonstrating perioperative mortality around 1%, reduced transfusion requirements, and shorter ICU and hospital stays, with consistently low complication rates across different minimally invasive approaches.^23^, ^24^, ^25^ These findings underscore the safety, efficiency, and reproducibility of contemporary MICS-AVR in experienced centers. Long-term data also support its durability: a single-center, 10-year experience with right anterior minithoracotomy showed low perioperative morbidity and favorable late outcomes, with low reoperation and late mortality rates.^20^ Furthermore, in an Asian cohort directly comparing MICS-AVR and transfemoral TAVI, MICS-AVR was found to be a feasible and efficient alternative, with trend toward fewer heart failure hospitalizations and PPM implantation—underscoring its competitive clinical performance in contemporary practice.^19^ From an economic standpoint, these clinical advantages—shorter ICU and ward stays, reduced transfusion rates, and faster recovery—translate into lower periprocedural costs, particularly in high-volume, experienced centers. Collectively, MICS-AVR represents a relevant contemporary surgical benchmark when assessing the cost-effectiveness of TAVI.

### Clinical and economic implications

Among patients with isolated severe AS at low surgical risk, TAVI and MICS-AVR demonstrated broadly comparable cost-effectiveness under current clinical and economic conditions. Although TAVI offered slightly greater health benefits at higher procedural costs, the resulting ICER was close to the conventional Japanese WTP threshold, indicating that its economic value may be considered borderline under present pricing structures.

The probabilistic sensitivity analysis further highlighted substantial decision uncertainty, with approximately one-half of simulations favoring TAVI at commonly cited WTP thresholds. From a payer or policy perspective, such findings suggest that reimbursement decisions may reasonably depend on long-term durability, procedural cost trajectories, and evolving real-world outcomes rather than on a single point estimate of cost-effectiveness.

Both strategies represent less invasive alternatives to full-sternotomy surgery, and their relative value depends largely on postoperative quality of life, device cost, and institutional expertise. Notably, the clinical and economic performance of MICS-AVR is strongly influenced by surgical experience and institutional volume, underscoring the importance of procedural standardization and team proficiency when interpreting its value relative to TAVI.

### Study limitations and future perspectives

This study has some limitations. First, the CEA was based on a decision analytical model that simplifies the complex clinical course of AS. Although model parameters were derived from a broad synthesis of published evidence—including randomized trials, meta-analyses, and large observational registries—the results remain subject to assumptions and approximations inherent to literature-based modeling. In particular, long-term transition probabilities in the chronic phase were assumed to be independent of prior perioperative events. In clinical practice, patients with stroke or heart failure may experience higher subsequent risks; however, robust state-specific transition data applicable to low-risk populations are limited. Similarly, in the probabilistic sensitivity analysis, model parameters were sampled independently, and potential correlations among clinical events, mortality, and health-related quality of life were not explicitly modeled due to the absence of reliable covariance data. This simplification may underestimate overall parameter uncertainty. Differences in study design, population characteristics, and health care systems may also have influenced estimated probabilities and utilities.

Second, long-term postoperative utility was identified as the most influential parameter in the model. Beyond 1 year, postoperative utility was assumed to remain stable in the absence of robust longitudinal data describing long-term quality-of-life trajectories in contemporary low-risk populations. Japan-specific quality-of-life data for contemporary low-risk AS patients undergoing TAVI or MICS-AVR remain limited, and utility inputs were therefore derived from international literature. Differences in health perception and recovery patterns across populations may influence absolute utility estimates. Furthermore, alternative long-term utility trajectories (eg, gradual decline over time) were not explicitly modeled, which may represent an additional source of structural uncertainty. Accordingly, the absence of domestic utility data represents a key limitation of this study, and further accumulation of Japan-specific patient-reported outcome data is warranted to refine future economic evaluations. Nevertheless, because identical methodologic assumptions were applied to both treatment arms, the relative comparison between TAVI and MICS-AVR is unlikely to change materially.

Third, cost estimates were primarily derived from the Japanese Diagnosis Procedure Combination database and national reimbursement schedules, representing the health care payer’s perspective rather than institutional or societal costs. As such, results reflect payer-level economics and may differ from hospital or societal cost structures.

Fourth, uncertainty persists regarding the long-term durability of contemporary TAVI prostheses. Although early and midterm outcomes of new-generation valves are favorable, data beyond 10 years are limited. Potential differences in valve longevity between TAVI and surgical bioprostheses could meaningfully affect lifetime cost-effectiveness.

Fifth, procedural outcomes and cost structures may vary among institutions, particularly for MICS-AVR, whose performance depends strongly on surgical experience, procedural volume, and institutional expertise. Therefore, caution is warranted when extrapolating these findings to centers with limited MICS experience.

Finally, this analysis reflects current device prices and clinical outcomes. As technologies evolve, reductions in device cost, improved valve durability, and enhanced procedural efficiency may shift the cost-effectiveness balance between TAVI and MICS-AVR in the future.

## Conclusions

TAVI and MICS-AVR showed broadly comparable cost-effectiveness in low-risk patients with isolated severe AS, with relative value largely determined by the performance of MICS-AVR, which is strongly influenced by institutional experience and surgical expertise.

## Funding Support and Author Disclosures

This work was supported by JSPS KAKENHI (grant number JP20K23226). Dr Kawakami has received lecture fees from Medtronic, Boston Scientific, Biotronik, Nihon Lifeline Co, Ltd, Abbott Japan, Philips Japan, Daiichi-Sankyo Co, Ltd, Pfizer Inc, and Boehringer Ingelheim. Dr Miyoshi has received lecture fees from Edwards Lifesciences. Dr Nishimura has received lecture fees from Edwards Lifesciences and Medtronic. All other authors have reported that they have no relationships relevant to the contents of this paper to disclose.
