# Supplementary material for: Cost-Effectiveness of TAVI vs Minimally Invasive SAVR for Low-Risk Aortic Stenosis in Japan
Source: JACC Asia. 2026 Apr 25;6(8):1386–98. doi: 10.1016/j.jacasi.2026.03.008 (PMC13458805; doi:10.1016/j.jacasi.2026.03.008)
Supplement: Supplementary file 1 [file mmc1.docx]

**Supplementary Table S1. Health-state utility parameters applied in the base-case and scenario analyses.**

| **Parameter** | **TAVI**  **Mean (range)** | **MICS-AVR**  **Mean (range)** | **Distribution** | **Source** |
| --- | --- | --- | --- | --- |
| Prior to intervention | 0.62 (0.50 to 0.70) | 0.62 (0.50 to 0.70) | Beta | ^1,2^ |
| Post-operative state (<1 year) | 0.78 (0.70 to 0.85) | 0.75 (0.68 to 0.83) | Beta | ^1,3^ |
| Post-operative state (≥1 year) | 0.80 (0.72 to 0.86) | 0.80 (0.72 to 0.86) | Beta | ^1,4^ |
| Death | 0 | 0 | Fixed | — |
| Chronic post-stroke sequelae (utility decrement) | −0.20(−0.10 to −0.30) | −0.20(−0.10 to −0.30) | Beta | ^5^ |
| Additional utility decrement during the first year after stroke | −0.10(−0.05 to −0.20) | −0.10(−0.05 to −0.20) | Beta | ^1,6^ |
| Disutility of heart failure hospitalization | −0.18(−0.11 to −0.26) | −0.18(−0.11 to −0.26) | Beta | ^6^ |
| Disutility of chronic heart failure | −0.08 (−0.04 to −0.12) | −0.08 (−0.04 to −0.12) | Beta | ^7,8^ |
| Disutility of pacemaker implantation | −0.02 (0 to −0.05) | −0.02 (0 to −0.05) | Beta | ^9,10^ |
| Disutility of SVD | −0.04 (0 to −0.09) | −0.04 (0 to −0.09) | Beta | ^1-4^ |

Abbreviations: AS = aortic stenosis; AVR = aortic valve replacement; MICS = minimally invasive cardiac surgery; TAVI = transcatheter aortic valve implantation; SVD = structural valve deterioration.

**Supplementary Table S2.** **Event probabilities applied in the scenario analysis.**

| **Parameter** | **TAVI**  **Mean (range)** | **MICS-AVR Japan**  **Mean (range)** | **Distribution** | **Source** |
| --- | --- | --- | --- | --- |
| **Peri-operative (30-day) complications** |  |  |  |  |
| Stroke | 0.6% (0.5–1.2%) | 0.8% (0.6–1.0%) | Beta | ^11-19^ |
| Permanent pacemaker implantation | 12.0% (6.5–17.4%) | 3.8% (2.5%–5.0%) | Beta | ^11-20^ |
| Major bleeding | 2.4% (2.4–3.6%) | 3.0% (3.0%–6.0%) | Beta | ^11-19^ |
| Reoperation | 2.0% (1.0–3.0%) | 0.3% (0.1-1.0%) | Beta | ^11-19^ |
| Death | 0.8% (0.5–1.3%) | 0.5% (0.3–0.7%) | Beta | ^11-19^ |
| **Chronic phase (annual) events** |  |  |  |  |
| All-cause death (per year) | 1.8% (1.5–2.2%) | 1.8% (1.2–2.6%) | Beta | ^11,12,16-18,21^ |
| Heart failure hospitalization (per year) | 1.6% (1.3–1.9%) | 1.0% (0.5–1.8%) | Beta | ^11-19^ |
| Permanent pacemaker implantation (per year) | 0.50% (0.30–0.80%) | 0.37% (0.22–0.60%) | Beta | ^11-20,22,23^ |
| Stroke (per year) | 0.64% (0.50–0.85%) | 0.83% (0.60–1.10%) | Beta | ^11-19^ |
| SVD (per year) | 1.7% (1.3–2.0%) | 0.4% (0.2–1.0%) | Beta | ^11-19,21,24,25^ |
| Reintervention for SVD (per year) | 0.63% (0.30–1.10%) | 0.10% (0.00–0.16%) | Beta | ^11-19,21,24,25^ |

Abbreviations: TAVI = transcatheter aortic valve implantation; MICS-AVR = minimally invasive surgical aortic valve replacement; SVD = structural valve deterioration.

**Supplementary Table S3. Base-case cost-effectiveness results by time horizon (1–15 years).**

| **Time horizon**  **(years)** | **Strategy** | **Total costs**  **(JPY)** | **Incremental costs**  **(JPY)** | **Total QALYs** | **Incremental QALYs** | **ICER**  **(JPY/QALY gained)** |
| --- | --- | --- | --- | --- | --- | --- |
| 1 year | MICS-AVR | 5,314,832 |  | 0.76 |  |  |
|  | TAVI | 6,423,625 | 1,108,794 | 0.77 | 0.02 | 73,598,664 |
| 2 years | MICS-AVR | 5,591,494 |  | 1.51 |  |  |
|  | TAVI | 6,663,112 | 1,071,618 | 1.53 | 0.02 | 48,965,859 |
| 3 years | MICS-AVR | 5,865,322 |  | 2.22 |  |  |
|  | TAVI | 6,899,237 | 1,033,915 | 2.25 | 0.03 | 33,655,351 |
| 4 years | MICS-AVR | 6,136,005 |  | 2.90 |  |  |
|  | TAVI | 7,131,900 | 995,896 | 2.94 | 0.04 | 24,098,788 |
| 5 years | MICS-AVR | 6,403,269 |  | 3.56 |  |  |
|  | TAVI | 7,361,014 | 957,745 | 3.61 | 0.05 | 17,910,251 |
| 6 years | MICS-AVR | 6,666,878 |  | 4.18 |  |  |
|  | TAVI | 7,586,504 | 919,626 | 4.25 | 0.07 | 13,733,265 |
| 7 years | MICS-AVR | 6,926,631 |  | 4.78 |  |  |
|  | TAVI | 7,808,307 | 881,677 | 4.86 | 0.08 | 10,804,157 |
| 8 years | MICS-AVR | 7,182,354 |  | 5.35 |  |  |
|  | TAVI | 8,026,373 | 844,019 | 5.45 | 0.10 | 8,680,523 |
| 9 years | MICS-AVR | 7,433,902 |  | 5.89 |  |  |
|  | TAVI | 8,240,660 | 806,758 | 6.01 | 0.11 | 7,096,238 |
| 10 years | MICS-AVR | 7,681,154 |  | 6.42 |  |  |
|  | TAVI | 8,451,136 | 769,982 | 6.55 | 0.13 | 5,885,068 |
| 11 years | MICS-AVR | 7,924,013 |  | 6.92 |  |  |
|  | TAVI | 8,657,779 | 733,766 | 7.07 | 0.15 | 4,939,500 |
| 12 years | MICS-AVR | 8,162,399 |  | 7.40 |  |  |
|  | TAVI | 8,860,574 | 698,174 | 7.56 | 0.17 | 4,187,780 |
| 13 years | MICS-AVR | 8,396,252 |  | 7.85 |  |  |
|  | TAVI | 9,059,512 | 663,260 | 8.04 | 0.19 | 3,580,674 |
| 14 years | MICS-AVR | 8,625,529 |  | 8.29 |  |  |
|  | TAVI | 9,254,594 | 629,065 | 8.49 | 0.20 | 3,083,548 |
| 15 years | MICS-AVR | 8,850,199 |  | 8.71 |  |  |
|  | TAVI | 9,445,824 | 595,625 | 8.93 | 0.22 | 2,671,501 |

Abbreviations: TAVI = transcatheter aortic valve implantation; MICS-AVR = minimally invasive surgical aortic valve replacement; QALY = quality-adjusted life year; ICER = incremental cost-effectiveness ratio; JPY = Japanese yen.

**Supplementary Table S4. Scenario analysis of cost-effectiveness results (10-year horizon).**

| **Strategy** | **Total costs**  **(JPY)** | **Incremental costs**  **(JPY)** | **Total QALYs** | **Incremental QALYs** | **ICER**  **(JPY/QALY gained)** |
| --- | --- | --- | --- | --- | --- |
| MICS-AVR | 6,670,307 |  | 6.60 |  |  |
| TAVI | 8,451,136 | 1,780,829 | 6.55 | -0.05 | Dominated |

Abbreviations: TAVI = transcatheter aortic valve implantation; MICS-AVR = minimally invasive surgical aortic valve replacement; QALY = quality-adjusted life year; ICER = incremental cost-effectiveness ratio; JPY = Japanese yen.

**Supplementary Figure S1. Cost-effectiveness acceptability curves in the Japanese high-volume center scenario.**

The probability of each strategy being cost-effective is plotted across a range of willingness-to-pay (WTP) thresholds. In the scenario reflecting outcomes from Japanese high-volume surgical centers, minimally invasive surgical aortic valve replacement (MICS-AVR) consistently showed higher acceptability than transcatheter aortic valve implantation (TAVI) within the conventional Japanese WTP range (5,000,000 JPY per quality-adjusted life-year [QALY]), indicating that MICS-AVR was more likely to be cost-effective under these optimized surgical conditions.

Abbreviations: MICS-AVR = minimally invasive surgical aortic valve replacement; TAVI = transcatheter aortic valve implantation; WTP = willingness-to-pay; QALY = quality-adjusted life-year.

**Supplementary Figure S2. Probabilistic sensitivity analysis (PSA) results in the Japanese high-volume center scenario.**

(A, B) Incremental cost-effectiveness scatterplots derived from 10,000 Monte Carlo simulations at willingness-to-pay (WTP) thresholds of 5,000,000 JPY (A) and 7,000,000 JPY (B) per quality-adjusted life-year (QALY). Each dot represents one simulation comparing minimally invasive surgical aortic valve replacement (MICS-AVR, blue) and transcatheter aortic valve implantation (TAVI, red). The green ellipse denotes the 95% confidence region of the simulated distribution. In this scenario, reflecting outcomes from Japanese high-volume surgical centers, MICS-AVR was cost-effective in 71.5% and 77.6% of iterations at WTP thresholds of 5,000,000 and 7,000,000 JPY per QALY, respectively.

Abbreviations: MICS-AVR = minimally invasive surgical aortic valve replacement; TAVI = transcatheter aortic valve implantation; PSA = probabilistic sensitivity analysis; WTP = willingness-to-pay; QALY = quality-adjusted life-year.

**References**

1.	Reynolds MR, Magnuson EA, Wang K, Thourani VH, Williams M, Zajarias A, Rihal CS, Brown DL, Smith CR, Leon MB, et al. Health-related quality of life after transcatheter or surgical aortic valve replacement in high-risk patients with severe aortic stenosis: results from the PARTNER (Placement of AoRTic TraNscathetER Valve) Trial (Cohort A). *J Am Coll Cardiol*. 2012;60:548-558. doi: 10.1016/j.jacc.2012.03.075

2.	Baron SJ, Wang K, House JA, Magnuson EA, Reynolds MR, Makkar R, Herrmann HC, Kodali S, Thourani VH, Kapadia S, et al. Cost-Effectiveness of Transcatheter Versus Surgical Aortic Valve Replacement in Patients With Severe Aortic Stenosis at Intermediate Risk. *Circulation*. 2019;139:877-888. doi: 10.1161/circulationaha.118.035236

3.	Galper BZ, Chinnakondepalli KM, Wang K, Magnuson EA, Lu M, Thourani VH, Kodali S, Makkar R, Herrmann HC, Kapadia S, et al. Economic Outcomes of Transcatheter Versus Surgical Aortic Valve Replacement in Patients with Severe Aortic Stenosis and Low Surgical Risk: Results from the PARTNER 3 Trial. *Circulation*. 2023;147:1594-1605. doi: 10.1161/circulationaha.122.062481

4.	Eerdekens R, Kats S, Grutters JP, Green M, Shore J, Candolfi P, Oortwijn W, Harst PV, Tonino P. Cost-utility analysis of TAVI compared with surgery in patients with severe aortic stenosis at low risk of surgical mortality in the Netherlands. *Cost Eff Resour Alloc*. 2024;22:24. doi: 10.1186/s12962-024-00531-6

5.	Joundi RA, Adekanye J, Leung AA, Ronksley P, Smith EE, Rebchuk AD, Field TS, Hill MD, Wilton SB, Bresee LC. Health State Utility Values in People With Stroke: A Systematic Review and Meta-Analysis. *J Am Heart Assoc*. 2022;11:e024296. doi: 10.1161/jaha.121.024296

6.	Keng MJ, Leal J, Bowman L, Armitage J, Mihaylova B. Decrements in health-related quality of life associated with adverse events in people with diabetes. *Diabetes Obes Metab*. 2022;24:530-538. doi: 10.1111/dom.14610

7.	Juenger J, Schellberg D, Kraemer S, Haunstetter A, Zugck C, Herzog W, Haass M. Health related quality of life in patients with congestive heart failure: comparison with other chronic diseases and relation to functional variables. *Heart*. 2002;87:235-241. doi: 10.1136/heart.87.3.235

8.	Di Tanna GL, Urbich M, Wirtz HS, Potrata B, Heisen M, Bennison C, Brazier J, Globe G. Health State Utilities of Patients with Heart Failure: A Systematic Literature Review. *PharmacoEconomics*. 2021;39:211-229. doi: 10.1007/s40273-020-00984-6

9.	Inácio NA, Muniz MN, Menezes ADSJ, Fernandes JF, Barbosa VA, Laranjeira TA, Arruda M. Health-Related Quality of Life in Elderly Patients with Pacemakers. *Braz J Cardiovasc Surg*. 2021;36:639-647. doi: 10.21470/1678-9741-2020-0522

10.	Lopez-Villegas A, Catalan-Matamoros D, Robles-Musso E, Peiro S. Effectiveness of pacemaker tele-monitoring on quality of life, functional capacity, event detection and workload: The PONIENTE trial. *Geriatr Gerontol Int*. 2016;16:1188-1195. doi: 10.1111/ggi.12612

11.	Smith CR, Leon MB, Mack MJ, Miller DC, Moses JW, Svensson LG, Tuzcu EM, Webb JG, Fontana GP, Makkar RR, et al. Transcatheter versus surgical aortic-valve replacement in high-risk patients. *The New England journal of medicine*. 2011;364:2187-2198. doi: 10.1056/NEJMoa1103510

12.	Reardon MJ, Van Mieghem NM, Popma JJ, Kleiman NS, Søndergaard L, Mumtaz M, Adams DH, Deeb GM, Maini B, Gada H, et al. Surgical or Transcatheter Aortic-Valve Replacement in Intermediate-Risk Patients. *The New England journal of medicine*. 2017;376:1321-1331. doi: 10.1056/NEJMoa1700456

13.	Glauber M, Miceli A, Gilmanov D, Ferrarini M, Bevilacqua S, Farneti PA, Solinas M. Right anterior minithoracotomy versus conventional aortic valve replacement: a propensity score matched study. *J Thorac Cardiovasc Surg*. 2013;145:1222-1226. doi: 10.1016/j.jtcvs.2012.03.064

14.	Merk DR, Lehmann S, Holzhey DM, Dohmen P, Candolfi P, Misfeld M, Mohr FW, Borger MA. Minimal invasive aortic valve replacement surgery is associated with improved survival: a propensity-matched comparison. *Eur J Cardiothorac Surg*. 2015;47:11-17; discussion 17. doi: 10.1093/ejcts/ezu068

15.	Gilmanov D, Bevilacqua S, Murzi M, Cerillo AG, Gasbarri T, Kallushi E, Miceli A, Glauber M. Minimally invasive and conventional aortic valve replacement: a propensity score analysis. *Ann Thorac Surg*. 2013;96:837-843. doi: 10.1016/j.athoracsur.2013.04.102

16.	Masuda T, Nakamura Y, Ito Y, Kuroda M, Nishijima S, Okuzono Y, Hirano T, Hori T. The learning curve of minimally invasive aortic valve replacement for aortic valve stenosis. *Gen Thorac Cardiovasc Surg*. 2020;68:565-570. doi: 10.1007/s11748-019-01234-z

17.	Furutachi A, Nakamura Y, Niitsuma K, Ushijima M, Yasumoto Y, Yoshiyama D, Kuroda M, Nakamae K, Hayashi Y, Nakayama T, et al. Midterm Outcomes of Minimally Invasive Aortic Valve Replacement via Right Lateral Minithoracotomy. *Innovations (Phila)*. 2025;20:48-56. doi: 10.1177/15569845241308005

18.	Japanese Cardiovascular Surgery Database (JCVSD). Nationwide outcomes of isolated aortic valve replacement in patients aged 65–79 years. 2017–2019 report.

19.	Kaneko T, Vemulapalli S, Kohsaka S, Shimamura K, Stebbins A, Kumamaru H, Nelson AJ, Kosinski A, Maeda K, Bavaria JE, et al. Practice Patterns and Outcomes of Transcatheter Aortic Valve Replacement in the United States and Japan: A Report From Joint Data Harmonization Initiative of STS/ACC TVT and J-TVT. *J Am Heart Assoc*. 2022;11:e023848. doi: 10.1161/jaha.121.023848

20.	Glaser N, Persson M, Dalén M, Sartipy U. Long-term Outcomes Associated With Permanent Pacemaker Implantation After Surgical Aortic Valve Replacement. *JAMA Netw Open*. 2021;4:e2116564. doi: 10.1001/jamanetworkopen.2021.16564

21.	Thyregod HGH, Ihlemann N, Jørgensen TH, Nissen H, Kjeldsen BJ, Petursson P, Chang Y, Franzen OW, Engstrøm T, Clemmensen P, et al. Five-Year Clinical and Echocardiographic Outcomes From the NOTION Randomized Clinical Trial in Patients at Lower Surgical Risk. *Circulation*. 2019;139:2714-2723. doi: 10.1161/circulationaha.118.036606

22.	Fadahunsi OO, Olowoyeye A, Ukaigwe A, Li Z, Vora AN, Vemulapalli S, Elgin E, Donato A. Incidence, Predictors, and Outcomes of Permanent Pacemaker Implantation Following Transcatheter Aortic Valve Replacement: Analysis From the U.S. Society of Thoracic Surgeons/American College of Cardiology TVT Registry. *JACC Cardiovasc Interv*. 2016;9:2189-2199. doi: 10.1016/j.jcin.2016.07.026

23.	Faroux L, Chen S, Muntané-Carol G, Regueiro A, Philippon F, Sondergaard L, Jørgensen TH, Lopez-Aguilera J, Kodali S, Leon M, et al. Clinical impact of conduction disturbances in transcatheter aortic valve replacement recipients: a systematic review and meta-analysis. *Eur Heart J*. 2020;41:2771-2781. doi: 10.1093/eurheartj/ehz924

24.	Bourguignon T, Bouquiaux-Stablo AL, Candolfi P, Mirza A, Loardi C, May MA, El-Khoury R, Marchand M, Aupart M. Very long-term outcomes of the Carpentier-Edwards Perimount valve in aortic position. *Ann Thorac Surg*. 2015;99:831-837. doi: 10.1016/j.athoracsur.2014.09.030

25.	Johnston DR, Soltesz EG, Vakil N, Rajeswaran J, Roselli EE, Sabik JF, 3rd, Smedira NG, Svensson LG, Lytle BW, Blackstone EH. Long-term durability of bioprosthetic aortic valves: implications from 12,569 implants. *Ann Thorac Surg*. 2015;99:1239-1247. doi: 10.1016/j.athoracsur.2014.10.070
